# Supplementary material for: The Trivalent Recombinant Chimeric Proteins Containing Immunodominant Fragments of Toxoplasma gondii SAG1 and SAG2 Antigens in Their Core—A Good Diagnostic Tool for Detecting IgG Antibodies in Human Serum Samples
Source: Int J Mol Sci. 2025 Jun 12;26(12):5621. doi: 10.3390/ijms26125621 (PMC12193361; doi:10.3390/ijms26125621)
Supplement: Supplementary file 1 [file ijms-26-05621-s001.zip › ijms-3659125-supplementary.pdf]

Table S1. Characteristics of recombinant plasmids containing fusion genes encoding recombinant chimeric proteins. Apart from SS-GRA5S, data from Ferra, B.T. et. al. The Development of *Toxoplasma gondii* Recombinant Trivalent Chimeric Proteins as an Alternative to *Toxoplasma* Lysate Antigen (TLA) in Enzyme-Linked Immunosorbent Assay (ELISA) for the Detection of Immunoglobulin G (IgG) in Small Ruminants. Int. J. Mol. Sci. 2024, 25, 4384. https://doi.org/10.3390/ijms25084384

Recombinant plasmid	Plasmid size [bp]	Gene fragment	Additional gene fragment	Encoding amino acid residues	GenBank Accession No.	GenPept Accession No.	Nucleotide
pET30/SAG1-SAG2-AMA1	8163	*sag1* (amino acid residues from 49-310); GeneBank Accession No. S76248.1; nucleotide from 453-1244 *sag2* (amino acid from 30-170); GeneBank Accession No. M33572.1; nucleotide from 269-691*/269-692**/269-693***	*ama1*	from 67-568	XM_002364813.1	XP_002364854.1	727-2232
pET30/SAG1-SAG2-AMA1S	7908		*ama1*	from 67-483	XM_002364813.1	XP_002364854.1	728-1979
pET30/SAG1-SAG2-GRA1	7158		*gra1*	from 24-190	M26007.1	AAA30141.1	681-1181
pET30/SAG1-SAG2-GRA2	7062		*gra2*	from 51-185	M99392.1	AAB59210.1	1128-1532
pET30/SAG1-SAG2-GRA5	6942		*gra5*	from 26-120	L06091.1	AAB63601.1	391-675
pET30/SAG1-SAG2-GRA5S	6864		*gra5*	from 26-94	L06091.1	AAB63601.1	392-598
pET30/SAG1-SAG2-GRA6	7257		*gra6*	from 30-228	L33814.1	AAC37235.1	506-1106
pET30/SAG1-SAG2-GRA7	7287		*gra7*	from 27-236	Y13863.1	CAA74178.1	156-785
pET30/SAG1-SAG2-GRA9	7551		*gra9*	from 21-318	AY371455.1	AAR10290.1	2148-2498 and 3116-3658
pET30/SAG1-SAG2-LDH2	7632		*ldh2*	from 2-326	XM_002368447.2	XP_002368488.1	909-1884
pET30/SAG1-SAG2-MAG1	7926		*mag1*	from 30-452	XM_002365659.1	XP_002365700.1	374-1643
pET30/SAG1-SAG2-MAG1S	7236		*mag1s*	from 30-222	XM_002365659.1	XP_002365700.1	374-954
pET30/SAG1-SAG2-MIC1	7953		*mic1*	from 25-456	Z71786.1	CAA96466.1	216-217, 537-1009, 1401-1479, and 1742-2483
pET30/SAG1-SAG2-MIC1S	7131		*mic1ex2*	from 25-182	Z71786.1	CAA96466.1	217 and 537-1010
pET30/SAG1-SAG2-MIC3	7536		*mic3*	from 67-359	AJ132530.1	CAB56644.1	894-1772
pET30/SAG1-SAG2-P35	7716		*p35*	from 26-377	AF310261.1	AAG32058.1	120-1178
pET30/SAG1-SAG2-P35S	7092		*p35*	from 26-170	AF310261.1	AAG32058.1	121-555
pET30/SAG1-SAG2-ROP1	7593		*rop1*	from 85-396	M71274.1	AAA69859.1	453-1388
pET30/SAG1-SAG2	6657	*sag1*	-	from 49-310	S76248.1	AAB33440.1	453-1244
		sag2	-	from 30-170	M33572.1	AAA30144.1	269-692

Explanation of symbols: * - in case of pET30/SAG1-SAG2-AMA1N, pET30/SAG1-SAG2-GRA5S, pET30/SAG1-SAG2-LDH2, pET30/SAG1-SAG2-MIC3, pET30/SAG1-SAG2-ROP1; ** - in case of pET30/SAG1-SAG2-AMA1, pET30/SAG1-SAG2-GRA1, pET30/SAG1-SAG2-GRA5, pET30/SAG1-SAG2-GRA6, pET30/SAG1-SAG2-GRA7, pET30/SAG1-SAG2-GRA9, pET30/SAG1-SAG2-MAG1, pET30/SAG1-SAG2-MAG1S, pET30/SAG1-SAG2-MIC1, pET30/SAG1-SAG2-MIC1S, pET30/SAG1-SAG2-P35; *** - in case of pET30/SAG1-SAG2-GRA2, pET30/SAG1-SAG2-P35S

Table S2. Characteristics of recombinant chimeric proteins. Apart from SS-GRA5S, data from Ferra, B.T. et. al. The Development of *Toxoplasma gondii* Recombinant Trivalent Chimeric Proteins as an Alternative to *Toxoplasma* Lysate Antigen (TLA) in Enzyme-Linked Immunosorbent Assay (ELISA) for the Detection of Immunoglobulin G (IgG) in Small Ruminants. Int. J. Mol. Sci. 2024, 25, 4384. https://doi.org/10.3390/ijms25084384

Recombinant chimeric protein	Amino acid residues	Additional immunodominant fragment	Amino acid residues	Number of amino acid residues	Mw [kDa]	pI
SAG1-SAG2-AMA1	SAG1 amino acid residues from 49-310<br>SAG2 amino acid residues from 30-170	AMA1	from 67-568	983	106.08	5.78
SAG1-SAG2-AMA1S		AMA1S	from 67-483	898	96.76	5.97
SAG1-SAG2-GRA1		GRA1	from 24-190	648	68.05	5.00
SAG1-SAG2-GRA2		GRA2	from 51-185	616	64.84	6.60
SAG1-SAG2-GRA5		GRA5	from 26-120	576	60.53	5.93
SAG1-SAG2-GRA5S		GRA5S	from 26-94	550	57.43	6.23
SAG1-SAG2-GRA6		GRA6	from 30-228	681	70.92	5.75
SAG1-SAG2-GRA7		GRA7	from 27-236	691	73.38	5.61
SAG1-SAG2-GRA9		GRA9	from 21-318	779	83.43	5.69
SAG1-SAG2-LDH2		LDH2	from 2-326	806	85.33	6.26
SAG1-SAG2-MAG1		MAG1	from 30-452	904	96.34	5.24
SAG1-SAG2-MAG1S		MAG1S	from 30-222	674	70.82	5.09
SAG1-SAG2-MIC1		MIC1	from 25-456	913	96.30	5.71
SAG1-SAG2-MIC1S		MIC1ex2	from 25-182	639	67.75	6.75
SAG1-SAG2-MIC3		MIC3	from 67-359	774	81.09	6.09
SAG1-SAG2-P35		P35	from 26-377	834	88.13	8.98
SAG1-SAG2-P35S		P35S	from 26-170	626	65.28	6.28
SAG1-SAG2-ROP1		ROP1	from 85-396	793	83.69	6.07
SAG1-SAG2		-	-	481	50.15	6.15

SAG1-SAG2 (SAG1 49-310 AA; SAG2 30-170 AA)

1 MHHHHHHSSG LVPRGSGMKE TAAAKFERQH MDSPDPDPPL VANQVVTCPD KKSTAAVILT
61 PTENHFTLKC PKTALTEPPT LAYSPNRQIC PAGTTSSCTS KAVTLSSLIP EAEDSWWTGD
121 SASLDTAGIK LTVPIEKFPV TTQTFVVGCI KGDDAQSCMV TVTVQARASS VVNNVARCSY
181 GADSTLGPVK LSAEGPTTMT LVCGKDGVKV PQDNNQYCSG TTLTGCNEKS FKDILPKLTE
241 NPWQGNASSD KGATLTIKKE AFPAESKSVI IGCTGGSPEK HHCTVKLEFA GAAGSAKSAE
301 TPAPIECTAG ATKTVDAPSS GSVVFQCGDK LTISPSGEGD VFYGKECTDS RKLTTVLPGA
361 VLTAKVQQPA KGPATYTLSY DGTPEKPQVL CYKCVAEAGA PAGRNNDGSS APTPKDCKLI
421 VRVPGADGRV TSGFDPVSLT DLGTDDDDKS PGFSSTMAIS DPNSSSVDKL AAALEHHHHH
481 H

SAG1-SAG2-AMA1 (SAG1 49-310 AA; SAG2 30-170 AA; AMA1 67-568 AA)

1 MHHHHHHSSG LVPRGSGMKE TAAAKFERQH MDSPDPDPPL VANQVVTCPD KKSTAAVILT
61 PTENHFTLKC PKTALTEPPT LAYSPNRQIC PAGTTSSCTS KAVTLSSLIP EAEDSWWTGD
121 SASLDTAGIK LTVPIEKFPV TTQTFVVGCI KGDDAQSCMV TVTVQARASS VVNNVARCSY
181 GADSTLGPVK LSAEGPTTMT LVCGKDGVKV PQDNNQYCSG TTLTGCNEKS FKDILPKLTE
241 NPWQGNASSD KGATLTIKKE AFPAESKSVI IGCTGGSPEK HHCTVKLEFA GAAGSAKSAE
301 TPAPIECTAG ATKTVDAPSS GSVVFQCGDK LTISPSGEGD VFYGKECTDS RKLTTVLPGA
361 VLTAKVQQPA KGPATYTLSY DGTPEKPQVL CYKCVAEAGA PAGRNNDGSS APTPKDCKLI
421 VRVPGADGRV TSGFDPVSLT DLGTDDDDKS PGFSSTMAIT SGNPFQANVE MKTFMERFNL
481 THHHQSGIYV DLGQDKEVDG TLYREPAGLC PIWGKHIELQ QPDRPPYRNN FLEDVPTEKE
541 YKQSGNPLPG GFNLNFVTPS GQRISPFPME LLEKNSNIKA STDLGRCAEF AFKTVAMDKN
601 NKATKYRYPF VYDSKKRLCH ILYVSMQLME GKKYCSVKGE PPDLTWYCFK PRKSVTENHH
661 LIYGSAYVGE NPDAFISKCP NQALRGYRFG VWKKGRCLDY TELTDTVIER VESKAQCWVK
721 TFENDGVASD QPHTYPLTSQ ASWNDWWPLH QSDQPHSGGV GRNYGFYYVD TTGEGKCALS
781 DQVPDCLVSD SAAVSYTAAG SLSEETPNFI IPSNPSVTPP TPETALQCTA DKFPDSFGAC
841 DVQACKRQKT SCVGGQIQST SVDCTADEQN ECGSNTALIA GLAVGGVLLL ALLGGGCYFA
901 KRLDRNKGVQ AAHHEHEFQS DRGARKKRPS DLMQEAEPSF WDEAEENIEQ DGETHVMVEG
961 ESDPNSSSVD KLAAALEHHH HHH

SAG1-SAG2-AMA1S (SAG1 49-310 AA; SAG2 30-170 AA; AMA1N 67-483 AA)

1 MHHHHHHSSG LVPRGSGMKE TAAAKFERQH MDSPDPDPPL VANQVVTCPD KKSTAAVILT
61 PTENHFTLKC PKTALTEPPT LAYSPNRQIC PAGTTSSCTS KAVTLSSLIP EAEDSWWTGD
121 SASLDTAGIK LTVPIEKFPV TTQTFVVGCI KGDDAQSCMV TVTVQARASS VVNNVARCSY
181 GADSTLGPVK LSAEGPTTMT LVCGKDGVKV PQDNNQYCSG TTLTGCNEKS FKDILPKLTE
241 NPWQGNASSD KGATLTIKKE AFPAESKSVI IGCTGGSPEK HHCTVKLEFA GAAGSAKSAE
301 TPAPIECTAG ATKTVDAPSS GSVVFQCGDK LTISPSGEGD VFYGKECTDS RKLTTVLPGA
361 VLTAKVQQPA KGPATYTLSY DGTPEKPQVL CYKCVAEAGA PAGRNNDGSS APTPKDCKLI
421 VRVPGADGRV TSGFDPVSLT TSGNPFQANV EMKTFMERFN LTHHHQSGIY VDLGQDKEVD
481 GTLYREPAGL CPIWGKHIEL QQPDRPPYRN NFLEDVPTEK EYKQSGNPLP GGFNLNFVTP
541 SGQRISPFPM ELLEKNSNIK ASTDLGRCAE FAFKTVAMDK NNKATKYRYP FVYDSKKRLC
601 HILYVSMQLM EGKKYCSVKG EPPDLTWYCF KPRKSVTENH HLIYGSAYVG ENPDAFISKC
661 PNQALRGYRF GVWKKGRCLD YTELTDTVIE RVESKAQCWV KTFENDGVAS DQPHTYPLTS
721 QASWNDWWPL HQSDQPHSGG VGRNYGFYYV DTTGEGKCAL SDQVPDCLVS DSAAVSYTAA
781 GSLSEETPNF IIPSNPSVTP PTPETALQCT ADKFPDSFGA CDVQACKRQK TSCVGGQIQS
841 TSVDCTADEQ NECGSNTDLG TDDDDKSPGF SSTMAISDPN SSSVDKLAAA LEHHHHHH

SAG1-SAG2-GRA1 (SAG1 49-310 AA; SAG2 30-170 AA; GRA1 24-190 AA)

1 MHHHHHHSSG LVPRGSGMKE TAAAKFERQH MDSPDPDPPL VANQVVTCPD KKSTAAVILT
61 PTENHFTLKC PKTALTEPPT LAYSPNRQIC PAGTTSSCTS KAVTLSSLIP EAEDSWWTGD
121 SASLDTAGIK LTVPIEKFPV TTQTFVVGCI KGDDAQSCMV TVTVQARASS VVNNVARCSY
181 GADSTLGPVK LSAEGPTTMT LVCGKDGVKV PQDNNQYCSG TTLTGCNEKS FKDILPKLTE
241 NPWQGNASSD KGATLTIKKE AFPAESKSVI IGCTGGSPEK HHCTVKLEFA GAAGSAKSAE
301 TPAPIECTAG ATKTVDAPSS GSVVFQCGDK LTISPSGEGD VFYGKECTDS RKLTTVLPGA
361 VLTAKVQQPA KGPATYTLSY DGTPEKPQVL CYKCVAEAGA PAGRNNDGSS APTPKDCKLI
421 VRVPGADGRV TSGFDPVSLT DLGTDDDDKS PGFSSTMAIA AEGGDNQSSA VSDRASLFGL
481 LSGGTGQGLG IGESVDLEMM GNTYRVERPT GNPDLLKIAI KASDGSYSEV GNVNVEEVID

541 TMKSMQRDED IFLRALNKGE TVEEAIEDVA QAEGLNSEQT LQLEDAVSAV ASVVQDEMKV
601 IDDVQQLEKD KQQLKDDIGF LTGERESDPN SSSVDKLAAA LEHHHHHH

SAG1-SAG2-GRA2 (SAG1 49-310 AA; SAG2 30-170 AA; GRA2 51-185 AA)
1 MHHHHHHSSG LVPRGSGMKE TAAAKFERQH MDSPDPDPPL VANQVVTCPD KKSTAAVILT
61 PTENHFTLKC PKTALTEPPT LAYSPNRQIC PAGTTSSCTS KAVTLSSLIP EAEDSWWTGD
121 SASLDTAGIK LTVPIEKFPV TTQTFVVGCI KGDDAQSCMV TVTVQARASS VVNNVARCSY
181 GADSTLGPVK LSAEGPTTMT LVCGKDGVKV PQDNNQYCSG TTLTGCNEKS FKDILPKLTE
241 NPWQGNASSD KGATLTIKKE AFPAESKSVI IGCTGGSPEK HHCTVKLEFA GAAGSAKSAE
301 TPAPIECTAG ATKTVDAPSS GSVVFQCGDK LTISPSGEGD VFYGKECTDS RKLTTVLPGA
361 VLTAKVQQPA KGPATYTLSY DGTPEKPQVL CYKCVAEAGA PAGRNNDGSS APTPKDCKLI
421 VRVPGADGRV TSGFDPVSLT GKGEHTPPLP DERQQEPEEP VSQRASRVAE QLFRKFLKFA
481 ENVGHHSEKA FKKAKVVAEK GFTAAKTHTV RGFKVAKEAA GRGMVTVGKK LANVESDRST
541 TTTQAPDSPN GLAETEVPVE PQQRAAHVPV PDFSQDLGTD DDDKSPGFSS TMAISDPNSS
601 SVDKLAAALE HHHHHH

SAG1-SAG2-GRA5 (SAG1 49-310 AA; SAG2 30-170 AA; GRA5 26-120 AA)
1 MHHHHHHSSG LVPRGSGMKE TAAAKFERQH MDSPDPDPPL VANQVVTCPD KKSTAAVILT
61 PTENHFTLKC PKTALTEPPT LAYSPNRQIC PAGTTSSCTS KAVTLSSLIP EAEDSWWTGD
121 SASLDTAGIK LTVPIEKFPV TTQTFVVGCI KGDDAQSCMV TVTVQARASS VVNNVARCSY
181 GADSTLGPVK LSAEGPTTMT LVCGKDGVKV PQDNNQYCSG TTLTGCNEKS FKDILPKLTE
241 NPWQGNASSD KGATLTIKKE AFPAESKSVI IGCTGGSPEK HHCTVKLEFA GAAGSAKSAE
301 TPAPIECTAG ATKTVDAPSS GSVVFQCGDK LTISPSGEGD VFYGKECTDS RKLTTVLPGA
361 VLTAKVQQPA KGPATYTLSY DGTPEKPQVL CYKCVAEAGA PAGRNNDGSS APTPKDCKLI
421 VRVPGADGRV TSGFDPVSLT DLGTDDDDKS PGFSSTMAIG STRDVGSGGD DSEGARGREQ
481 QQVQQHEQNE DRSLFERGRA AVTGHPVRTA VGLAAAVVAV VSLLRLLKRR RRRAIQEESK
541 ESATAEEEEV AEEESDPNSS SVDKLAAALE HHHHHH

SAG1-SAG2-GRA5S (SAG1 49-310 AA; SAG2 30-170 AA; GRA5 26-94 AA)
1 MHHHHHHSSG LVPRGSGMKE TAAAKFERQH MDSPDPDPPL VANQVVTCPD KKSTAAVILT
61 PTENHFTLKC PKTALTEPPT LAYSPNRQIC PAGTTSSCTS KAVTLSSLIP EAEDSWWTGD
121 SASLDTAGIK LTVPIEKFPV TTQTFVVGCI KGDDAQSCMV TVTVQARASS VVNNVARCSY
181 GADSTLGPVK LSAEGPTTMT LVCGKDGVKV PQDNNQYCSG TTLTGCNEKS FKDILPKLTE
241 NPWQGNASSD KGATLTIKKE AFPAESKSVI IGCTGGSPEK HHCTVKLEFA GAAGSAKSAE
301 TPAPIECTAG ATKTVDAPSS GSVVFQCGDK LTISPSGEGD VFYGKECTDS RKLTTVLPGA
361 VLTAKVQQPA KGPATYTLSY DGTPEKPQVL CYKCVAEAGA PAGRNNDGSS APTPKDCKLI
421 VRVPGADGRV TSGFDPVSLT GSTRDVGSGG DDSEGARGRE QQQVQQHEQN EDRSLFERGR
481 AAVTGHPVRT AVGLAAAVVA VVSLLRLLKD LGTDDDDKSP GFSSTMAISD PNSSSVDKLA
541 AALEHHHHHH

SAG1-SAG2-GRA6 (SAG1 49-310 AA; SAG2 30-170 AA; GRA6 30-228 AA)
1 MHHHHHHSSG LVPRGSGMKE TAAAKFERQH MDSPDPDPPL VANQVVTCPD KKSTAAVILT
61 PTENHFTLKC PKTALTEPPT LAYSPNRQIC PAGTTSSCTS KAVTLSSLIP EAEDSWWTGD
121 SASLDTAGIK LTVPIEKFPV TTQTFVVGCI KGDDAQSCMV TVTVQARASS VVNNVARCSY
181 GADSTLGPVK LSAEGPTTMT LVCGKDGVKV PQDNNQYCSG TTLTGCNEKS FKDILPKLTE
241 NPWQGNASSD KGATLTIKKE AFPAESKSVI IGCTGGSPEK HHCTVKLEFA GAAGSAKSAE
301 TPAPIECTAG ATKTVDAPSS GSVVFQCGDK LTISPSGEGD VFYGKECTDS RKLTTVLPGA
361 VLTAKVQQPA KGPATYTLSY DGTPEKPQVL CYKCVAEAGA PAGRNNDGSS APTPKDCKLI
421 VRVPGADGRV TSGFDPVSLT DLGTDDDDKS PGFSSTMAIM GVLVNSLGGV RVAADSGGVK
481 QTPSETGSSG GQQEAVGTTE DYVNSSAMGG GQGDSLAEDD TTSEAAEGDV DPFPVLANEG
541 KSEARGPSLE ERIEEQGTRR RYSSVQEPQA KVPCKRTQKR HRLIGAVVLA VSVAMLTAFF
601 LRRTGRRSPQ EPSGDGGGND AGNNAGNGGN EGRGYGGRGE GGAEDDRRPL HPERVNVFES
661 DPNSSSVDKL AAALEHHHHH H

SAG1-SAG2-GRA7 (SAG1 49-310 AA; SAG2 30-170 AA; GRA7 27-236 AA)
1 MHHHHHHSSG LVPRGSGMKE TAAAKFERQH MDSPDPDPPL VANQVVTCPD KKSTAAVILT
61 PTENHFTLKC PKTALTEPPT LAYSPNRQIC PAGTTSSCTS KAVTLSSLIP EAEDSWWTGD
121 SASLDTAGIK LTVPIEKFPV TTQTFVVGCI KGDDAQSCMV TVTVQARASS VVNNVARCSY
181 GADSTLGPVK LSAEGPTTMT LVCGKDGVKV PQDNNQYCSG TTLTGCNEKS FKDILPKLTE

241 NPWQGNASSD KGATLTIKKE AFPAESKSVI IGCTGGSPEK HHCTVKLEFA GAAGSAKSAE
301 TPAPIECTAG ATKTVDAPSS GSVVFQCGDK LTISPSGEGD VFYGKECTDS RKLTTVLPGA
361 VLTAKVQQPA KGPATYTLSY DGTPEKPQVL CYKCVAEAGA PAGRNNDGSS APTPKDCKLI
421 VRVPGADGRV TSGFDPVSLT DLGTDDDDKS PGFSSTMAMA TASDDELMSR IRNSDFFDGQ
481 APVDSLRPTN AGVDSKGTDD HLTTSMDKAS VESQLPRREP LETEPDEQEE VHFRKRGVRS
541 DAEVTDDNIY EEHTDRKVVP RKSEGKRSFK DLLKKLALPA VGMGASYFAA DRLVPELTEE
601 QQRGDEPLTT GQNVGTVLGF AALAAAAAFL GMGLTRTYRH FSPRKNRSRQ PALEQEVPES
661 GEDGEDARQS DPNSSSVDKL AAALEHHHHH H

SAG1-SAG2-GRA9 (SAG1 49-310 AA; SAG2 30-170 AA; GRA9 21-318 AA)
1 MHHHHHHSSG LVPRGSGMKE TAAAKFERQH MDSPDPDPPL VANQVVTCPD KKSTAAVILT
61 PTENHFTLKC PKTALTEPPT LAYSPNRQIC PAGTTSSCTS KAVTLSSLIP EAEDSWWTGD
121 SASLDTAGIK LTVPIEKFPV TTQTFVVGCI KGDDAQSCMV TVTVQARASS VVNNVARCSY
181 GADSTLGPVK LSAEGPTTMT LVCGKDGVKV PQDNNQYCSG TTLTGCNEKS FKDILPKLTE
241 NPWQGNASSD KGATLTIKKE AFPAESKSVI IGCTGGSPEK HHCTVKLEFA GAAGSAKSAE
301 TPAPIECTAG ATKTVDAPSS GSVVFQCGDK LTISPSGEGD VFYGKECTDS RKLTTVLPGA
361 VLTAKVQQPA KGPATYTLSY DGTPEKPQVL CYKCVAEAGA PAGRNNDGSS APTPKDCKLI
421 VRVPGADGRV TSGFDPVSLT DLGTDDDDKS PGFSSTMAIL DLFLGESGVY LFGKASESDV
481 ALKVPEDPVP EEPRREPEKH VDLFGEDWKQ FGGSGFGDFS KVEFENLFSQ VHEMMRRLMG
541 RGADGFGPSL LGDSPGFHFP RLRALQPKTK LEKTGTCQYV VTWAPEVTAE NVRVILHLQR
601 RQVEVQYRAA TRRDEKTEGG ESHSMSKEQS SQLMSVDPQC IMTREVVAQK LAGWTDNTHT
661 ATAGTPKKLL ISFPSPDHIK EMVKEGYLPD NALERVLAGD FEGFSRTQMC LVSGRNRTEC
721 AFAEGQEVEL EEKPLPSDSS PVTSVELPRL SQEDRGLSDP NSSSVDKLAA ALEHHHHHH

SAG1-SAG2-LDH2 (SAG1 49-310 AA; SAG2 30-170 AA; LDH2 2-326 AA)
1 MHHHHHHSSG LVPRGSGMKE TAAAKFERQH MDSPDPDPPL VANQVVTCPD KKSTAAVILT
61 PTENHFTLKC PKTALTEPPT LAYSPNRQIC PAGTTSSCTS KAVTLSSLIP EAEDSWWTGD
121 SASLDTAGIK LTVPIEKFPV TTQTFVVGCI KGDDAQSCMV TVTVQARASS VVNNVARCSY
181 GADSTLGPVK LSAEGPTTMT LVCGKDGVKV PQDNNQYCSG TTLTGCNEKS FKDILPKLTE
241 NPWQGNASSD KGATLTIKKE AFPAESKSVI IGCTGGSPEK HHCTVKLEFA GAAGSAKSAE
301 TPAPIECTAG ATKTVDAPSS GSVVFQCGDK LTISPSGEGD VFYGKECTDS RKLTTVLPGA
361 VLTAKVQQPA KGPATYTLSY DGTPEKPQVL CYKCVAEAGA PAGRNNDGSS APTPKDCKLI
421 VRVPGADGRV TSGFDPVSLT TGTVSRRKKI AMIGSGMIGG TMGYLCVLRE LADVVLFDVV
481 TGMPEGKALD DSQATSIADT NVSVTSANQY EKIAGSDVVI ITAGLTKVPG KSDKEWSRND
541 LLPFNAKIIR EVAQGVKKYC PLAFVIVVTN PLDCMVKCFH EASGLPKNMV CGMANVLDSA
601 RFRRFIADQL EISPRDIQAT VIGTHGDHML PLARYVTVNG FPLREFIKKG KMTEAKLAEI
661 VERTKKAGGE IVRLLGQGSA YYAPALSAIT MAQAFLKDEK RVLPCSVYCQ GEYGLHDMFI
721 GLPAVIGGGG IEQVIELELT HEEQECFRKS VDDVVELNKS LAALGDLGTD DDDKSPGFSS
781 TMAISDPNSS SVDKLAAALE HHHHHH

SAG1-SAG2-MAG1 (SAG1 49-310 AA; SAG2 30-170 AA; MAG1 30-452 AA)
1 MHHHHHHSSG LVPRGSGMKE TAAAKFERQH MDSPDPDPPL VANQVVTCPD KKSTAAVILT
61 PTENHFTLKC PKTALTEPPT LAYSPNRQIC PAGTTSSCTS KAVTLSSLIP EAEDSWWTGD
121 SASLDTAGIK LTVPIEKFPV TTQTFVVGCI KGDDAQSCMV TVTVQARASS VVNNVARCSY
181 GADSTLGPVK LSAEGPTTMT LVCGKDGVKV PQDNNQYCSG TTLTGCNEKS FKDILPKLTE
241 NPWQGNASSD KGATLTIKKE AFPAESKSVI IGCTGGSPEK HHCTVKLEFA GAAGSAKSAE
301 TPAPIECTAG ATKTVDAPSS GSVVFQCGDK LTISPSGEGD VFYGKECTDS RKLTTVLPGA
361 VLTAKVQQPA KGPATYTLSY DGTPEKPQVL CYKCVAEAGA PAGRNNDGSS APTPKDCKLI
421 VRVPGADGRV TSGFDPVSLT DLGTDDDDKS PGFSSTMAMS QRVPELPEVE SFDEVGTGAR
481 RSGSIATLLP QDAVLYENSE DVAVPSDSAS TPSYFHVESP SASVEAATGA VGEVVPDCEE
541 QQEQGDTTLS DHDFHSGGTE QEGLPETEVA HQHETEEQYG TEGMPPPVLP PAPVVHPRFI
601 AVPGPSVPVP FFSLPDIHPD QVVYILRVQG SGDFDISFEV GRAVKQLEAI KKAYREATGK
661 LEADELESER GPAVSPRRRL VDLIKDNQRR LRAALQKIKI QKKLEEIDDL LQLTRALKAM
721 DARLRACQDM APIEEALCHK TKAFGEMVSQ KAKEIREKAA SLSSLLGVDA VEKQLRRVEP
781 EHEDNTRVEA RVEELQKALE KAASEAKQLV GTAAGEIEEG VKADTQAVQD SSKDVLTKSQ
841 LALVEAFKAI QRALLEAKTK ELVEPTSKEA EEARQILAEQ AASDPNSSSV DKLAAALEHH
901 HHHH

SAG1-SAG2-MAG1S (SAG1 49-310 AA; SAG2 30-170 AA; MAG1S 30-222 AA)

```
  1 MHHHHHHSSG LVPRGSGMKE TAAAKFERQH MDSPDPDPPL VANQVVTCPD KKSTAAVILT
 61 PTENHFTLKC PKTALTEPPT LAYSPNRQIC PAGTTSSCTS KAVTLSSLIP EAEDSWWTGD
121 SASLDTAGIK LTVPIEKFPV TTQTFVVGCI KGDDAQSCMV TVTVQARASS VVNNVARCSY
181 GADSTLGPVK LSAEGPTTMT LVCGKDGVKV PQDNNQYCSG TTLTGCNEKS FKDILPKLTE
241 NPWQGNASSD KGATLTIKKE AFPAESKSVI IGCTGGSPEK HHCTVKLEFA GAAGSAKSAE
301 TPAPIECTAG ATKTVDAPSS GSVVFQCGDK LTISPSGEGD VFYGKECTDS RKLTTVLPGA
361 VLTAKVQQPA KGPATYTLSY DGTPEKPQVL CYKCVAEAGA PAGRNNDGSS APTPKDCKLI
421 VRVPGADGRV TSGFDPVSLT DLGTDDDDKS PGFSSTMAMS QRVPELPEVE SFDEVGTGAR
481 RSGSIATLLP QDAVLYENSE DVAVPSDSAS TPSYFHVESP SASVEAATGA VGEVVPDCEE
541 QQEQGDTTLS DHDFHSGGTE QEGLPETEVA HQHETEEQYG TEGMPPPVLP PAPVVHPRFI
601 AVPGPSVPVP FFSLPDIHPD QVVYILRVQG SGDFDISFEV GRAVKQLEAI KKSDPNSSSV
661 DKLAAALEHH HHHH
```

SAG1-SAG2-MIC1 (SAG1 49-310 AA; SAG2 30-170 AA; MIC1 25-456 AA)

```
  1 MHHHHHHSSG LVPRGSGMKE TAAAKFERQH MDSPDPDPPL VANQVVTCPD KKSTAAVILT
 61 PTENHFTLKC PKTALTEPPT LAYSPNRQIC PAGTTSSCTS KAVTLSSLIP EAEDSWWTGD
121 SASLDTAGIK LTVPIEKFPV TTQTFVVGCI KGDDAQSCMV TVTVQARASS VVNNVARCSY
181 GADSTLGPVK LSAEGPTTMT LVCGKDGVKV PQDNNQYCSG TTLTGCNEKS FKDILPKLTE
241 NPWQGNASSD KGATLTIKKE AFPAESKSVI IGCTGGSPEK HHCTVKLEFA GAAGSAKSAE
301 TPAPIECTAG ATKTVDAPSS GSVVFQCGDK LTISPSGEGD VFYGKECTDS RKLTTVLPGA
361 VLTAKVQQPA KGPATYTLSY DGTPEKPQVL CYKCVAEAGA PAGRNNDGSS APTPKDCKLI
421 VRVPGADGRV TSGFDPVSLT DLGTDDDDKS PGFSSTMAIA SHSHSPASGR YIQQMLDQRC
481 QEIAAELCQS GLRKMCVPSS RIVARNAVGI THQNTLQWRC FDTASLLESN QENNGVNCVD
541 DCGHTIPCPG GVHRQNSNHA TRHEILSKLV EEGVQRFCSP YQASANKYCN DKFPGTIARR
581 SKGFGNNVEV AWRCYEKASL LYSVYAECAS NCGTTWYCPG GRRGTSTELD KRHYTEEEGI
641 RQAIGSVDSP CSEVEVCLPK DENPPLCLDE SGQISRTGGG PPSQPPEMQQ PADRSDERGG
701 GKEQSPGGEA QPDHPTKGGN IDLPEKSTSP EKTPKTEIHG DSTKATLEEG QQLTLTFIST
761 KLDVAVGSCH SLVANFLDGF LKFQTGSNSA FDVVEVEEPA GPAVLTIGLG HKGRLAVVLD
821 YTRLNAALGS AAYVVEDSGC SSSEEVSFQG VGSGATLVVT TLGESPTAVS ASDPNSSSVD
881 KLAAALEHHH HHH
```

SAG1-SAG2-MIC1ex2 (SAG1 49-310 AA; SAG2 30-170 AA; MIC1ex2 25-182 AA)

```
  1 MHHHHHHSSG LVPRGSGMKE TAAAKFERQH MDSPDPDPPL VANQVVTCPD KKSTAAVILT
 61 PTENHFTLKC PKTALTEPPT LAYSPNRQIC PAGTTSSCTS KAVTLSSLIP EAEDSWWTGD
121 SASLDTAGIK LTVPIEKFPV TTQTFVVGCI KGDDAQSCMV TVTVQARASS VVNNVARCSY
181 GADSTLGPVK LSAEGPTTMT LVCGKDGVKV PQDNNQYCSG TTLTGCNEKS FKDILPKLTE
241 NPWQGNASSD KGATLTIKKE AFPAESKSVI IGCTGGSPEK HHCTVKLEFA GAAGSAKSAE
301 TPAPIECTAG ATKTVDAPSS GSVVFQCGDK LTISPSGEGD VFYGKECTDS RKLTTVLPGA
361 VLTAKVQQPA KGPATYTLSY DGTPEKPQVL CYKCVAEAGA PAGRNNDGSS APTPKDCKLI
421 VRVPGADGRV TSGFDPVSLT ASHSHSPASG RYIQQMLDQR CQEIAAELCQ SGLRKMCVPS
481 SRIVARNAVG ITHQNTLQWR CFDTASLLES NQENNGVNCV DDCGHTIPCP GGVHRQNSNH
541 ATRHEILSKL VEEGVQRFCS PYQASANKYC NDKFPGTIAR RSKGFGNNVE VAWRCYEKDL
601 GTDDDDKSPG FSSTMAISDP NSSSVDKLAA ALEHHHHHH
```

SAG1-SAG2-MIC3 (SAG1 49-310 AA; SAG2 30-170 AA; MIC3 67-359 AA)

```
  1 MHHHHHHSSG LVPRGSGMKE TAAAKFERQH MDSPDPDPPL VANQVVTCPD KKSTAAVILT
 61 PTENHFTLKC PKTALTEPPT LAYSPNRQIC PAGTTSSCTS KAVTLSSLIP EAEDSWWTGD
121 SASLDTAGIK LTVPIEKFPV TTQTFVVGCI KGDDAQSCMV TVTVQARASS VVNNVARCSY
181 GADSTLGPVK LSAEGPTTMT LVCGKDGVKV PQDNNQYCSG TTLTGCNEKS FKDILPKLTE
241 NPWQGNASSD KGATLTIKKE AFPAESKSVI IGCTGGSPEK HHCTVKLEFA GAAGSAKSAE
301 TPAPIECTAG ATKTVDAPSS GSVVFQCGDK LTISPSGEGD VFYGKECTDS RKLTTVLPGA
361 VLTAKVQQPA KGPATYTLSY DGTPEKPQVL CYKCVAEAGA PAGRNNDGSS APTPKDCKLI
421 VRVPGADGRV TSGFDPVSLT SPSKQETQLC AISSEGKPCR NRQLHTDNGY FIGASCPKSA
481 CCSKTMCGPG GCGEFCSSNW IFCSSSLIYH PDKSYGGDCS CEKQGHRCDK NAECVENLDA
541 GGGVHCKCKD GFVGTGLTCS EDPCSKRGNA KCGPNGTCIV VDSVSYTCTC GDGETLVNLP
601 EGGQGCKRTG CHAFRENCSP GRCIDDASHE NGYTCECPTG YSREVTSKAE ESCVEGVEVT
```

```
661 LAEKCEKEFG ISASSCKCDN GYSGSASATS HHGKGESGSE GSLSEKMNIV FKCPSGYHPR
721 YHAHTVTCEK IKQDLGTDDD DKSPGFSSTM AISDPNSSSV DKLAAALEHH HHHH
```

SAG1-SAG2-P35 (SAG1 49-310 AA; SAG2 30-170 AA; P35 26-377 AA)

```
  1 MHHHHHHSSG LVPRGSGMKE TAAAKFERQH MDSPDPDPPL VANQVVTCPD KKSTAAVILT
 61 PTENHFTLKC PKTALTEPPT LAYSPNRQIC PAGTTSSCTS KAVTLSSLIP EAEDSWWTGD
121 SASLDTAGIK LTVPIEKFPV TTQTFVVGCI KGDDAQSCMV TVTVQARASS VVNNVARCSY
181 GADSTLGPVK LSAEGPTTMT LVCGKDGVKV PQDNNQYCSG TTLTGCNEKS FKDILPKLTE
241 NPWQGNASSD KGATLTIKKE AFPAESKSVI IGCTGGSPEK HHCTVKLEFA GAAGSAKSAE
301 TPAPIECTAG ATKTVDAPSS GSVVFQCGDK LTISPSGEGD VFYGKECTDS RKLTTVLPGA
361 VLTAKVQQPA KGPATYTLSY DGTPEKPQVL CYKCVAEAGA PAGRNNDGSS APTPKDCKLI
421 VRVPGADGRV TSGFDPVSLT DLGTDDDDKS PGFSSTMAIG PLSYHPSSYG ASYPNPSNPL
481 HGMPKPENPV RPPPPGFHPS VIPNPPYPLG TPAGMPQPEV PPLQHPPPTG SPPAAAPQPP
541 YPVGTPVMPQ PEIPPVHRPP PPGFRPEVAP VPPYPVGTPT GMPQPEIPAV HHPFPYVTTT
601 TTAAPRVLVY KIPYGGAAPP RAPPVPPRMG PSDISTHVRG AIRRQPGTTT TTTSRKLLFR
661 TAVVAAMAAA LITLFRQRPV FMEGVRMFPN LHMPQPEIPA VHHPFPYVTT TTTAAPRVLV
721 YKIPYGGAAP PRAPPVPPRM GPSDISTHVR GAIRRQPGTT TTTTSRKLLF RTAVVAAMAA
781 ALITLFRQRP VFMEGVRMFP NLHYRFTVTT QKSDPNSSSV DKLAAALEHH HHHH
```

SAG1-SAG2-P35S (SAG1 49-310 AA; SAG2 30-170 AA; P35S 26-170 AA)

```
  1 MHHHHHHSSG LVPRGSGMKE TAAAKFERQH MDSPDPDPPL VANQVVTCPD KKSTAAVILT
 61 PTENHFTLKC PKTALTEPPT LAYSPNRQIC PAGTTSSCTS KAVTLSSLIP EAEDSWWTGD
121 SASLDTAGIK LTVPIEKFPV TTQTFVVGCI KGDDAQSCMV TVTVQARASS VVNNVARCSY
181 GADSTLGPVK LSAEGPTTMT LVCGKDGVKV PQDNNQYCSG TTLTGCNEKS FKDILPKLTE
241 NPWQGNASSD KGATLTIKKE AFPAESKSVI IGCTGGSPEK HHCTVKLEFA GAAGSAKSAE
301 TPAPIECTAG ATKTVDAPSS GSVVFQCGDK LTISPSGEGD VFYGKECTDS RKLTTVLPGA
361 VLTAKVQQPA KGPATYTLSY DGTPEKPQVL CYKCVAEAGA PAGRNNDGSS APTPKDCKLI
421 VRVPGADGRV TSGFDPVSLT GPLSYHPSSY GASYPNPSNP LHGMPKPENP VRPPPPGFHP
481 SVIPNPPYPL GTPAGMPQPE VPPLQHPPPT GSPPAAAPQP PYPVGTPVMP QPEIPPVHRP
541 PPPGFRPEVA PVPPYPVGTP TGMPQPEIPA VHHPFPYVTT TTTAADLGTD DDDKSPGFSS
601 TMAISDPNSS SVDKLAAALE HHHHHH
```

SAG1-SAG2-ROP1 (SAG1 49-310 AA; SAG2 30-170 AA; ROP1 85-396 AA)

```
  1 MHHHHHHSSG LVPRGSGMKE TAAAKFERQH MDSPDPDPPL VANQVVTCPD KKSTAAVILT
 61 PTENHFTLKC PKTALTEPPT LAYSPNRQIC PAGTTSSCTS KAVTLSSLIP EAEDSWWTGD
121 SASLDTAGIK LTVPIEKFPV TTQTFVVGCI KGDDAQSCMV TVTVQARASS VVNNVARCSY
181 GADSTLGPVK LSAEGPTTMT LVCGKDGVKV PQDNNQYCSG TTLTGCNEKS FKDILPKLTE
241 NPWQGNASSD KGATLTIKKE AFPAESKSVI IGCTGGSPEK HHCTVKLEFA GAAGSAKSAE
301 TPAPIECTAG ATKTVDAPSS GSVVFQCGDK LTISPSGEGD VFYGKECTDS RKLTTVLPGA
361 VLTAKVQQPA KGPATYTLSY DGTPEKPQVL CYKCVAEAGA PAGRNNDGSS APTPKDCKLI
421 VRVPGADGRV TSGFDPVSLT PVRGPDQVPA RGEAALVTEE TPAQQPAVAL GSAEGEGTST
481 TESASENSED DDTFHDALQE LPEDGLEVRP PNAQELPPPN VQELPPPTEQ ELPPSTEQEL
541 PPPVGEGQRL QVPGEHGPQG PPYDDQQLLL EPTEEQQEGP QEPLPPPPPP TRGEQPEGQQ
601 PQGPVRQNFF RRALGAARSR FGGARRHVSG VFRRVRGGLN RIVGGVRSGF RRAREGVVGG
661 VRRLTSGASL GLRRVGEGLR RSFYRVRGAV SSGRRRAADG ASNVRERFVA AGGRVRDAFG
721 AGLTRLRRRG RTNGEEGRPL LGEGREQDDG SQDLGTDDDD KSPGFSSTMA ISDPNSSSVD
781 KLAAALEHHH HHH
```

Figure S1. Amino acid sequences of recombinant chimeric proteins. Apart from SS-GRA5S, data from Ferra, B.T. et. al. The Development of *Toxoplasma gondii* Recombinant Trivalent Chimeric Proteins as an Alternative to *Toxoplasma* Lysate Antigen (TLA) in Enzyme-Linked Immunosorbent Assay (ELISA) for the Detection of Immunoglobulin G (IgG) in Small Ruminants. Int. J. Mol. Sci. 2024, 25, 4384. https://doi.org/10.3390/ijms25084384

Table S3. Oligonucleotide primers used for the amplification of gene fragments. Apart from SS-GRA5S, data from Ferra, B.T. et. al. The Development of *Toxoplasma gondii* Recombinant Trivalent Chimeric Proteins as an Alternative to *Toxoplasma* Lysate Antigen (TLA) in Enzyme-Linked Immunosorbent Assay (ELISA) for the Detection of Immunoglobulin G (IgG) in Small Ruminants. Int. J. Mol. Sci. 2024, 25, 4384. https://doi.org/10.3390/ijms25084384

Recombinant chimeric protein	Gene fragment	Primer sequence	Corresponding to protein residues
SAG1-SAG2-AMA1	*ama1*	SS-AMA1For 5′-CTCAACCATGGCGATCACGTCGGGGAATCCCTTTCA-3′<br>SS-AMA1Rev 5′-GAATTCGGATCCGATTCCCCCTCGACCATAACATGTG-3′	67-568
SAG1-SAG2-AMA1S	*sag1*	SS-SAG1For 5′-TGGACAGCCCAGATCCGGATCCCCCTCTTGTTGC-3′<br>SS-SAG1Rev 5′-TGGGCGCTGGCGTCTCAGCCGATTTTGCTGAC-3′	49-310
	sag2	SS-SAG2For 5′-GTCAGCAAAATCGGCTGAGACGCCAGCGCCCA-3′<br>SS-S2/AMA1For 5′-AGGGATTCCCCGACGTCGTGAGAGACACAGGG-3′	30-170
	ama1	SSA-AMA1For 5′-CCCTGTGTCTCTCACGACGTCGGGGAATCCCT-3′<br>SSA-AMA1Rev 5′-ATCGGTACCCAGATCAGTGTTAGAGCCACATTCATTTTGTTCG-3′	67-483
SAG1-SAG2-GRA1	*gra1*	SS-GRA1For 5′-CTCAACCATGGCGATCGCTGCCGAAGGCG-3′<br>SS-GRA1Rev 5′-GAATTCGGATCCGATTCTCTCTCTCCTGTTAGGAACCCAAT-3′	24-190
SAG1-SAG2-GRA2	*sag1*	SS-SAG1For 5′-TGGACAGCCCAGATCCGGATCCCCCTCTTGTTGC-3′<br>SS-SAG1Rev 5′-TGGGCGCTGGCGTCTCAGCCGATTTTGCTGAC-3′	49-310
	sag2	SS-SAG2For 5′-GTCAGCAAAATCGGCTGAGACGCCAGCGCCCA-3′<br>SS-S2/GRA2Rev 5′-GGTGTATGTTCACCTTTTCCCGTGAGAGACACAGGGTC-3′	30-170
	gra2	SSG-GRA2For 5′-GACCCTGTGTCTCTCACGGGAAAAGGTGAACATACACC-3′<br>SSG-GRA2Rev 5′-ATCGGTACCCAGATCCTGCGAAAAGTCTGGGACGG-3′	51-185
SAG1-SAG2-GRA5	*gra5*	SS-GRA5For 5′-CTCAACCATGGCGATCGGTTCAACGCGTGACG-3′<br>SS-GRA5Rev 5′-GAATTCGGATCCGATTCTTCCTCGGCAACTTCTTCCT-3′	26-120
SAG1-SAG2-GRA5S	*sag1*	SS-SAG1For 5′-TGGACAGCCCAGATCCGGATCCCCCTCTTGTTGC-3′<br>SS-SAG1Rev 5′-TGGGCGCTGGCGTCTCAGCCGATTTTGCTGAC-3′	49-310
	sag2	SS-SAG2For 5′-GTCAGCAAAATCGGCTGAGACGCCAGCGCCCA-3′<br>SS-S2/GRA5For 5′-GTCACGCGTTGAACCCGTGAGAGACACAGGG-3′	30-170
	gra5	SS-GRA5SFor 5′-CCCTGTGTCTCTCACGGGTTCAACGCGTGAC-3′<br>SS-GRA5SRev 5′-ATCGGTACCCAGATCTTTCAACAATCGCAGTAGTGACACAAC-3′	26-94
SAG1-SAG2-GRA6	*gra6*	SS-GRA6For 5′-CTCAACCATGGCGATCATGGGTGTACTCGTCAATTCGTTG-3′<br>SS-GRA6Rev 5′-GAATTCGGATCCGATTCAAACACATTCACACGTTCCGG-3′	30-228
SAG1-SAG2-GRA7	*gra7*	SS-GRA7For 5′-CTCAACCATGGCGATGGCCACCGCGTCAGAT-3′<br>SS-GRA7Rev 5′-GAATTCGGATCCGATTGGCGGGCATCCTCCC-3′	27-236
SAG1-SAG2-GRA9	*gra9*	SS-GRA9For 5′-CTCAACCATGGCGATACTCGACCTTTTCCTCGGTGAA-3′<br>SS-GRA9Rev 5′-GAATTCGGATCCGATAGTCCTCGGTCTTCCTGCG-3′	21-318
SAG1-SAG2-LDH2	*sag1*	SS-SAG1For 5′-TGGACAGCCCAGATCCGGATCCCCCTCTTGTTGC-3′<br>SS-SAG1Rev 5′-TGGGCGCTGGCGTCTCAGCCGATTTTGCTGAC-3′	49-310
	sag2	SS-SAG2For 5′-GTCAGCAAAATCGGCTGAGACGCCAGCGCCCA-3′<br>SSL-S2/LDH2Rev 5′-TGCTAACGGTACCCGTCGTGAGAGACACAGGG-3′	30-170
	ldh2	SSL-LDH2For 5′-CCCTGTGTCTCTCACGACGGGTACCGTTAGCA-3′<br>SSL-LDH2Rev 5′-ATCGGTACCCAGATCACCCAGCGCCGCT-3′	2-326
SAG1-SAG2-MAG1	*mag1*	SS-MAG1For 5′-CTCAACCATGGCGATGAGCCAAAGGGTGCCAGAG-3′<br>SS-MAG1Rev 5′-GAATTCGGATCCGATGCTGCCTGTTCCGCTAAGAT-3′	30-452
SAG1-SAG2-MAG1S	*mag1*	SS-MAG1For 5′-CTCAACCATGGCGATGAGCCAAAGGGTGCCAGAG-3′<br>SS-MAG1SRev 5′-GAATTCGGATCCGATTTCTTGATGGCTTCCAACTGCT-3′	30-222
SAG1-SAG2-MIC1	*mic1*	SS-MIC1For 5′-CTCAACCATGGCGATAGCGTCGCATTCTCATTCGC-3′<br>SS-MIC1Rev 5′-GAATTCGGATCCGATGCAGAGACGGCCGTAGG-3′	25-456
SAG1-SAG2-MIC1S	*sag1*	SS-SAG1For 5′-TGGACAGCCCAGATCCGGATCCCCCTCTTGTTGC-3′<br>SS-SAG1Rev 5′-TGGGCGCTGGCGTCTCAGCCGATTTTGCTGAC-3′	49-310
	sag2	SS-SAG2For 5′-GTCAGCAAAATCGGCTGAGACGCCAGCGCCCA-3′<br>SSM-S2/MIC1Rev 5′-CGAATGAGAATGCGACGCCGTGAGAGACACAGGGT-3′	30-170
	mic1	SSM-MIC1For 5′-ACCCTGTGTCTCTCACGGCGTCGCATTCTCATTCG-3′<br>SSM-MIC1Rev 5′-ATCGGTACCCAGATCCTTCTCGTAACACCTCCACGCA-3′	25-182
SAG1-SAG2-MIC3	*sag1*	SS-SAG1For 5′-TGGACAGCCCAGATCCGGATCCCCCTCTTGTTGC-3′<br>SS-SAG1Rev 5′-TGGGCGCTGGCGTCTCAGCCGATTTTGCTGAC-3′	49-310
	sag2	SS-SAG2For 5′-GTCAGCAAAATCGGCTGAGACGCCAGCGCCCA-3′<br>SSM-S2/MIC3Rev 5′-CCTGCTTGCTGGGGGACGTGAGAGACACAGG-3′	30-170

	mic3	SSM-MIC3For 5′-CCTGTGTCTCTCACGTCCCCCAGCAAGCAGG-3′<br>SSM-MIC3Rev 5′-ATCGGTACCCAGATCCTGCTTAATTTTCTCACACGTCACGG-3′	67-359
SAG1-SAG2-P35	*p35*	SS-P35For 5′-CTCAACCATGGCGATCGGTCCTTTGAGTTATCATCCAAGC-3′<br>SS-P35Rev 5′-GAATTCGGATCCGATTTCTGCGTCGTTACGGTGAATCT-3′	26-377
SAG1-SAG2-P35S	*sag1*	SS-SAG1For 5′-TGGACAGCCCAGATCCGGATCCCCCTCTTGTTGC-3′<br>SS-SAG1Rev 5′-TGGGCGCTGGCGTCTCAGCCGATTTTGCTGAC-3′	49-310
	sag2	SS-SAG2For 5′-GTCAGCAAAATCGGCTGAGACGCCAGCGCCCA-3′<br>SSP-S2/P35Rev 5′-GATGATAACTCAAAGGACCCGTGAGAGACACAGGGTC-3′	30-170
	p35	SSP-P35For 5′-GACCCTGTGTCTCTCACGGGTCCTTTGAGTTATCATC-3′<br>SSP-P35Rev 5′-ATCGGTACCCAGATCAGCAGCTGTCGTGGTTGT-3′	26-170
SAG1-SAG2-ROP1	*sag1*	SS-SAG1For 5′-TGGACAGCCCAGATCCGGATCCCCCTCTTGTTGC-3′<br>SS-SAG1Rev 5′-TGGGCGCTGGCGTCTCAGCCGATTTTGCTGAC-3′	49-310
	sag2	SS-SAG2For 5′-GTCAGCAAAATCGGCTGAGACGCCAGCGCCCA-3′<br>SSR-S2/ROP1Rev 5′-CGGGCCTCTGACAGGCGTGAGAGACACAGG-3′	30-170
	rop1	SSR-ROP1For 5′-CCTGTGTCTCTCACGCCTGTCAGAGGCCCG-3′<br>SSR-ROP1Rev 5′-ATCGGTACCCAGATCTTGCGATCCATCATCCTGCTCTC-3′	85-396
SAG1-SAG2	*sag1*	SS-SAG1For 5′-TGGACAGCCCAGATCCGGATCCCCCTCTTGTTGC-3′<br>SS-SAG1Rev 5′-TGGGCGCTGGCGTCTCAGCCGATTTTGCTGAC-3′	49-310
	sag2	SS-SAG2For 5′-GTCAGCAAAATCGGCTGAGACGCCAGCGCCCA-3′<br>SS-SAG2Rev 5′-ATCGGTACCCAGATCCGTGAGAGACACAGGGTCAAAC-3′	30-170

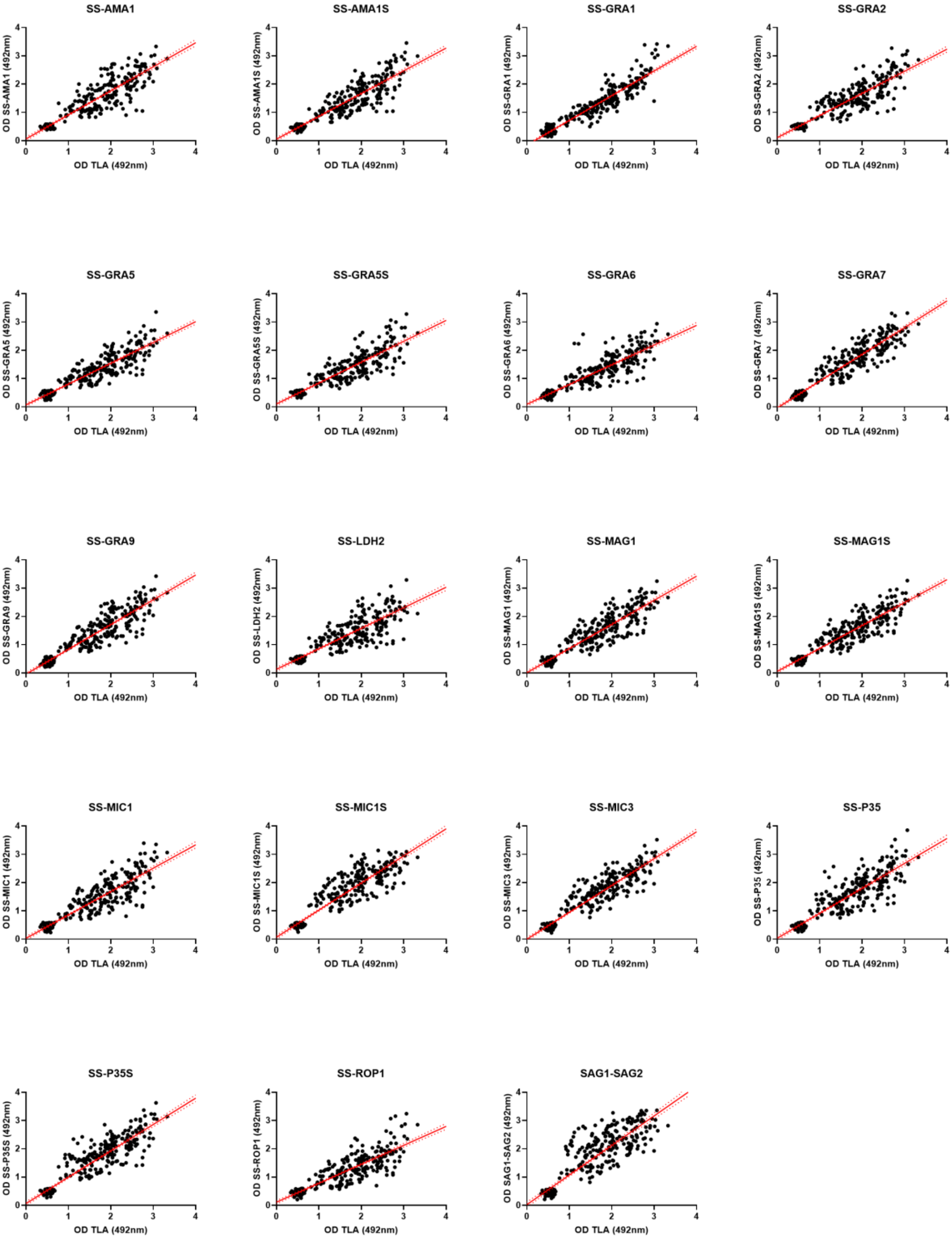

Figure S2. Scatter plot of TLA and antigens results for each individual, with linear regression fit and 95% CI.

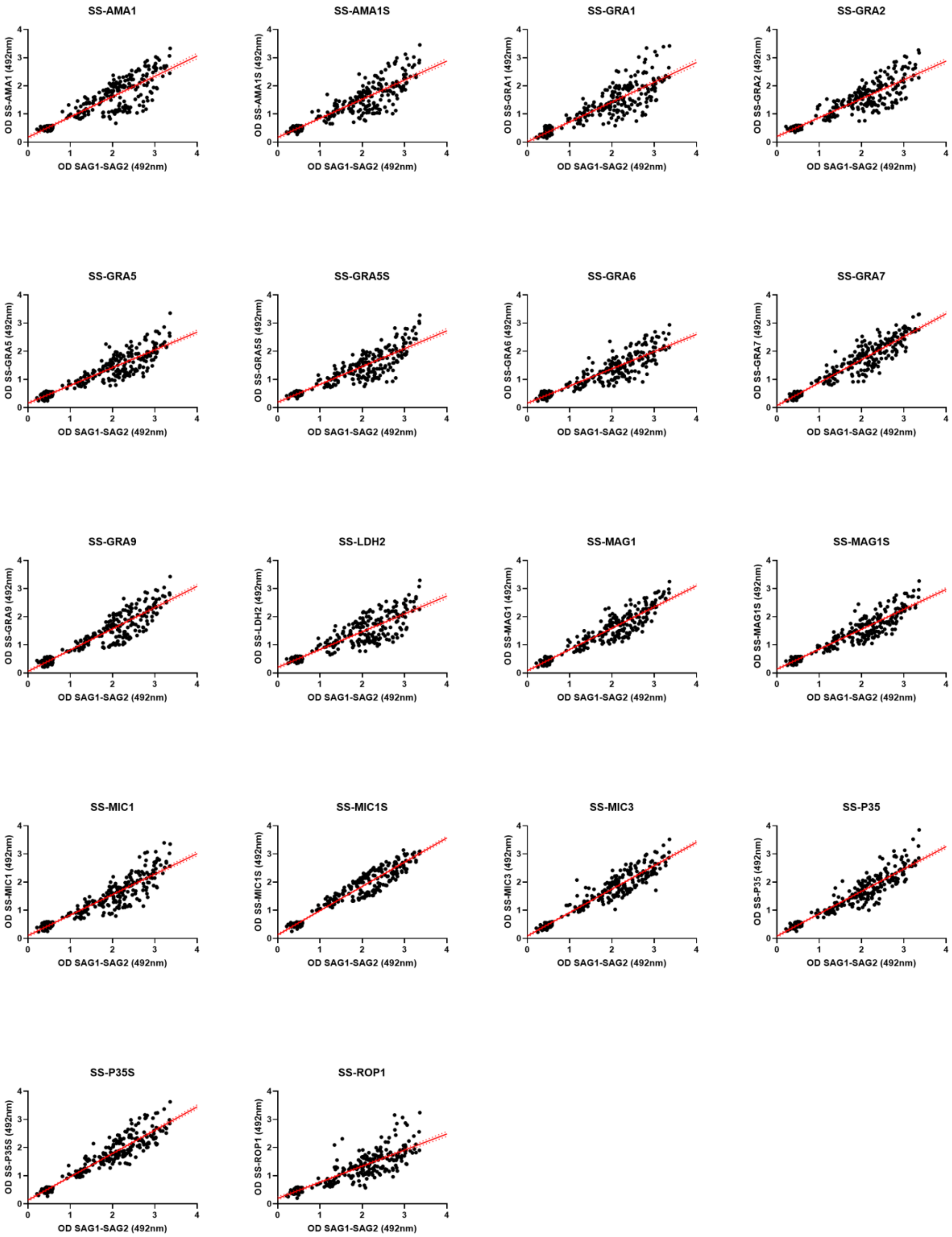

Figure S3. Scatter plot of SS and antigens results for each individual, with linear regression fit and 95% CI.

Table S4. The mean of the positive/negative ratio of human serum samples, split into groups, compared with RM one-way ANOVA, followed by Bonferroni post hoc, comparing every antigen preparation to TLA within the serum group. ANOVA p<0.0001, matching p<0.0001 in every serum group scenario.

**Antigen**	**Serum sample group**											
	**Acute n=54**		**Chronic IgG <200 IU/ml n=40**		**Chronic IgG 101-200 IU/ml n=30**		**Chronic IgG <100 IU/ml n=76**		**All chronic n=146**		**All positive n=200**	
	Mean	p-value	Mean	p-value	Mean	p-value	Mean	p-value	Mean	p-value	Mean	p-value
TLA	3.038	-	4.728	-	3.857	-	3.130	-	3.717	-	3.534	-
SS-AMA1FL	2.479	**<0.0001**	4.968	0.7799	3.893	>0.9999	3.191	>0.9999	3.822	>0.9999	3.459	>0.9999
SS-AMA1S	2.528	**<0.0001**	4.846	>0.9999	3.692	>0.9999	2.809	**0.0022**	3.548	0.1178	3.273	**<0.0001**
SS-GRA1	3.675	**<0.0001**	6.697	**<0.0001**	5.179	**<0.0001**	3.790	**<0.0001**	4.872	**<0.0001**	4.548	**<0.0001**
SS-GRA2	2.566	**0.0037**	4.683	>0.9999	3.495	0.1731	2.843	**0.0097**	3.481	**0.0016**	3.234	**<0.0001**
SS-GRA5FL	2.787	0.24	4.697	>0.9999	3.489	0.4128	2.956	0.5733	3.543	0.1009	3.339	**0.0044**
SS-GRA5S	2.551	**<0.0001**	4.526	>0.9999	3.185	**0.0009**	2.704	**<0.0001**	3.302	**<0.0001**	3.099	**<0.0001**
SS-GRA6	2.822	>0.9999	4.594	>0.9999	3.455	0.1244	2.627	**<0.0001**	3.336	**<0.0001**	3.197	**<0.0001**
SS-GRA7	3.550	**0.0003**	5.824	**<0.0001**	4.298	0.1142	3.580	**<0.0001**	4.343	**<0.0001**	4.129	**<0.0001**
SS-GRA9	3.231	>0.9999	5.665	**<0.0001**	4.448	**0.0462**	3.498	**0.0025**	4.287	**<0.0001**	4.002	**<0.0001**
SS-LDH2	2.432	**<0.0001**	4.570	>0.9999	3.101	**0.0006**	3.130	>0.9999	3.519	0.1328	3.225	**<0.0001**
SS-MAG1FL	3.351	0.1874	5.460	**<0.0001**	4.003	>0.9999	3.220	>0.9999	3.995	**0.0015**	3.821	**<0.0001**
SS-MAG1S	3.084	>0.9999	4.853	>0.9999	3.660	>0.9999	2.945	0.5398	3.615	>0.9999	3.472	>0.9999
SS-MIC1FL	2.977	>0.9999	5.314	**0.0004**	3.805	>0.9999	2.891	0.1374	3.743	>0.9999	3.536	>0.9999
SS-MIC1S	3.600	**0.0002**	5.089	0.0843	4.542	**0.0099**	3.631	**<0.0001**	4.217	**<0.0001**	4.051	**<0.0001**
SS-MIC3	3.785	**<0.0001**	5.598	**<0.0001**	4.505	**0.0009**	3.500	**0.0001**	4.281	**<0.0001**	4.147	**<0.0001**
SS-P35FL	3.423	0.2384	5.350	**0.0028**	3.966	>0.9999	3.124	>0.9999	3.907	0.1352	3.776	**0.0046**
SS-P35S	3.558	**0.0072**	5.075	0.1685	4.254	0.1444	3.241	>0.9999	3.952	**0.0023**	3.846	**<0.0001**
SS-ROP1	2.540	**0.0016**	4.312	0.118	3.207	**0.0029**	2.565	**<0.0001**	3.176	**<0.0001**	3.004	**<0.0001**
SAG1-SAG2	4.877	**<0.0001**	6.032	**<0.0001**	4.818	**0.0005**	3.677	**<0.0001**	4.557	**<0.0001**	4.643	**<0.0001**

Table S5. The mean of the positive/negative ratio of human serum samples, split into groups, compared with RM one-way ANOVA, followed by Bonferroni post hoc, **pairs of matching antigens** composed of either a full antigen or a shorter sequence, within the serum group. ANOVA p<0.0001, matching p<0.0001 in every serum group scenario.

**Antigen**	**Serum sample group**											
	**Acute n=54**		**Chronic IgG <200 IU/ml n=40**		**Chronic IgG 101-200 IU/ml n=30**		**Chronic IgG <100 IU/ml n=76**		**All chronic n=146**		**All positive n=200**	
	Mean	p-value	Mean	p-value	Mean	p-value	Mean	p-value	Mean	p-value	Mean	p-value
SS-AMA1FL	2.479	**-**	4.968	**-**	3.893	**-**	3.191	**-**	3.822	**-**	3.459	**-**
SS-AMA1S	2.528	0.7947	4.846	0.6967	3.692	0.4180	2.809	**<0.0001**	3.548	**<0.0001**	3.273	**<0.0001**
SS-GRA5FL	2.787	**-**	4.697	**-**	3.489	**-**	2.956	**-**	3.543	**-**	3.339	**-**
SS-GRA5S	2.551	**0.0064**	4.526	0.2446	3.185	**0.0002**	2.704	**<0.0001**	3.302	**<0.0001**	3.099	**<0.0001**
SS-MAG1FL	3.351	**-**	5.460	**-**	4.003	**-**	3.220	**-**	3.995	**-**	3.821	**-**
SS-MAG1S	3.084	**<0.0001**	4.853	**<0.0001**	3.66	**<0.0001**	2.945	**<0.0001**	3.615	**<0.0001**	3.472	**<0.0001**
SS-MIC1FL	2.977	**-**	5.314	**-**	3.805	**-**	2.891	**-**	3.743	**-**	3.536	**-**
SS-MIC1S	3.600	**<0.0001**	5.089	**0.0466**	4.542	**<0.0001**	3.631	**<0.0001**	4.217	**<0.0001**	4.051	**<0.0001**
SS-P35FL	3.423	**-**	5.350	**-**	3.966	**-**	3.124	**-**	3.907	**-**	3.776	**-**
SS-P35S	3.558	**0.0475**	5.075	**0.0001**	4.254	**0.0031**	3.241	**0.0034**	3.952	0.8437	3.846	0.0620

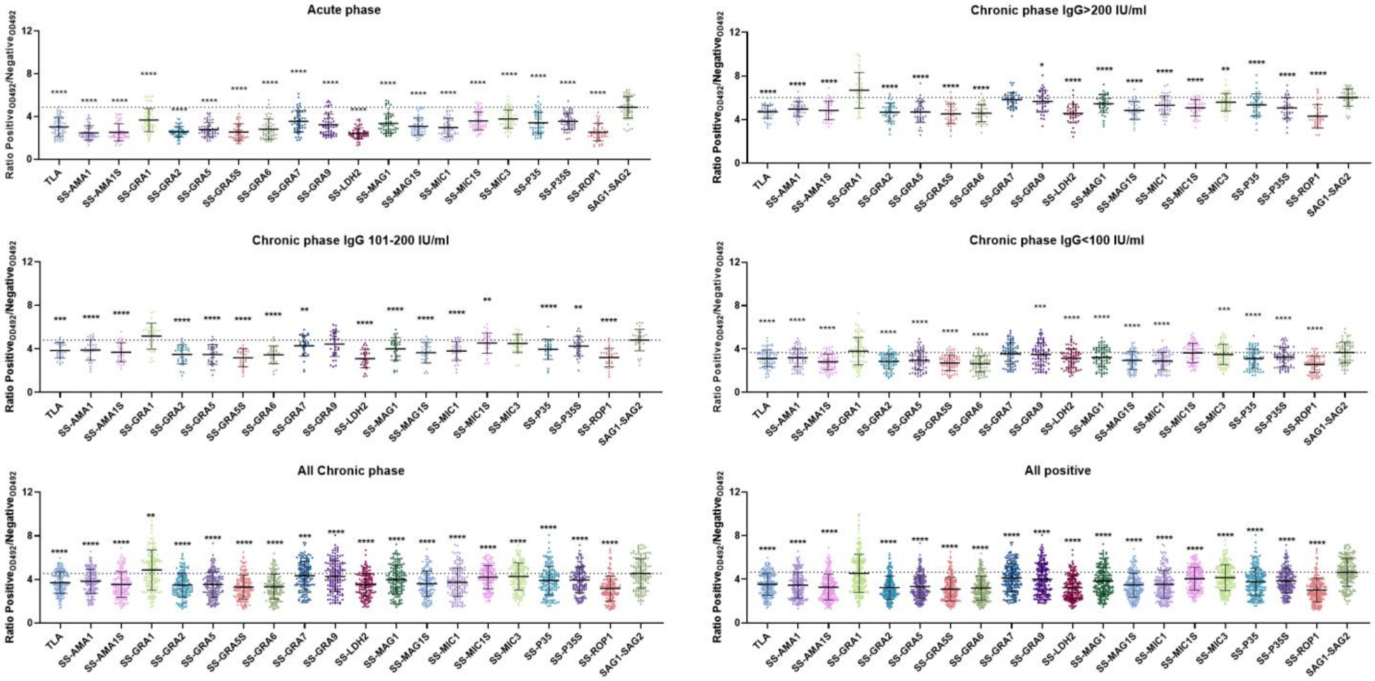

Figure S4. Comparison of immunoreactivity in the IgG ELISA positive/negative ratio. The ratio is calculated by dividing the value of a given sample by the average value of samples from non-infected people for the antigen used in the test. Analysis was performed using repeated measures one-way ANOVA, followed by Bonferroni`s multiple comparison test to compare each antigen with SAG1-SAG2. ns – $p_{adj} \geq 0.05$, (*) – $p_{adj} < 0.05$, (**) – $p_{adj} \leq 0.01$, (***) – $p_{adj} \leq 0.001$, (****) – $p_{adj} \leq 0.0001$. The data are presented as mean and standard deviation. The dotted line marks the mean of SAG1-SAG2.

Table S6. The mean of the positive/negative ratio of human serum samples, split into groups, compared with RM one-way ANOVA, followed by Bonferroni post-hoc, comparing every antigen preparation to **SAG1-SAG2** within the serum group. ANOVA p<0.0001, matching p<0.0001 in every serum group scenario.

Antigen	Serum sample group											
	**Acute n=54**		**Chronic IgG <200 IU/ml n=40**		**Chronic IgG 101-200 IU/ml n=30**		**Chronic IgG <100 IU/ml n=76**		**All chronic n=146**		**All positive n=200**	
	Mean	p-value	Mean	p-value	Mean	p-value	Mean	p-value	Mean	p-value	Mean	p-value
TLA	3.038	**<0.0001**	4.728	**<0.0001**	3.857	**0.0005**	3.130	**<0.0001**	3.717	**<0.0001**	3.534	**<0.0001**
SS-AMA1FL	2.479	**<0.0001**	4.968	**<0.0001**	3.893	**<0.0001**	3.191	**<0.0001**	3.822	**<0.0001**	3.459	**<0.0001**
SS-AMA1S	2.528	**<0.0001**	4.846	**<0.0001**	3.692	**<0.0001**	2.809	**<0.0001**	3.548	**<0.0001**	3.273	**<0.0001**
SS-GRA1	3.675	**<0.0001**	6.697	0.1069	5.179	>0.9999	3.790	>0.9999	4.872	**0.0072**	4.548	>0.9999
SS-GRA2	2.566	**<0.0001**	4.683	**<0.0001**	3.495	**<0.0001**	2.843	**<0.0001**	3.481	**<0.0001**	3.234	**<0.0001**
SS-GRA5FL	2.787	**<0.0001**	4.697	**<0.0001**	3.489	**<0.0001**	2.956	**<0.0001**	3.543	**<0.0001**	3.339	**<0.0001**
SS-GRA5S	2.551	**<0.0001**	4.526	**<0.0001**	3.185	**<0.0001**	2.704	**<0.0001**	3.302	**<0.0001**	3.099	**<0.0001**
SS-GRA6	2.822	**<0.0001**	4.594	**<0.0001**	3.455	**<0.0001**	2.627	**<0.0001**	3.336	**<0.0001**	3.197	**<0.0001**
SS-GRA7	3.55	**<0.0001**	5.824	0.6666	4.298	**0.0031**	3.580	0.9267	4.343	**0.0001**	4.129	**<0.0001**
SS-GRA9	3.231	**<0.0001**	5.665	**0.0329**	4.448	0.1407	3.498	**0.0006**	4.287	**<0.0001**	4.002	**<0.0001**
SS-LDH2	2.432	**<0.0001**	4.570	**<0.0001**	3.101	**<0.0001**	3.130	**<0.0001**	3.519	**<0.0001**	3.225	**<0.0001**
SS-MAG1FL	3.351	**<0.0001**	5.460	**<0.0001**	4.003	**<0.0001**	3.220	**<0.0001**	3.995	**<0.0001**	3.821	**<0.0001**
SS-MAG1S	3.084	**<0.0001**	4.853	**<0.0001**	3.660	**<0.0001**	2.945	**<0.0001**	3.615	**<0.0001**	3.472	**<0.0001**
SS-MIC1FL	2.977	**<0.0001**	5.314	**<0.0001**	3.805	**<0.0001**	2.891	**<0.0001**	3.743	**<0.0001**	3.536	**<0.0001**
SS-MIC1S	3.600	**<0.0001**	5.089	**<0.0001**	4.542	**0.009**	3.631	>0.9999	4.217	**<0.0001**	4.051	**<0.0001**
SS-MIC3	3.785	**<0.0001**	5.598	**0.0039**	4.505	0.8821	3.500	**0.0005**	4.281	**<0.0001**	4.147	**<0.0001**
SS-P35FL	3.423	**<0.0001**	5.350	**<0.0001**	3.966	**<0.0001**	3.124	**<0.0001**	3.907	**<0.0001**	3.776	**<0.0001**
SS-P35S	3.558	**<0.0001**	5.075	**<0.0001**	4.254	**0.0084**	3.241	**<0.0001**	3.952	**<0.0001**	3.846	**<0.0001**
SS-ROP1	2.540	**<0.0001**	4.312	**<0.0001**	3.207	**<0.0001**	2.565	**<0.0001**	3.176	**<0.0001**	3.004	**<0.0001**
SAG1-SAG2	4.877	-	6.032	-	4.818	-	3.677	-	4.557	-	4.643	-
